# Nickel(II) and Zinc(II) Binding to Tau Protein Fragments Containing Histidine and Cysteine

**DOI:** 10.1007/s12011-026-05162-x

**Published:** 2026-06-16

**Authors:** Faareha Mazhar, Noémi Piskolti, Viktória Kovács, Katalin Várnagy

**Affiliations:** 1 Department of Inorganic and Analytical Chemistry, University of Debrecen, Debrecen, H-4032 Hungary; 2 Doctoral School of Chemistry, University of Debrecen, Egyetem tér 1, Debrecen, H-4032 Hungary

**Keywords:** Cysteine, Histidine, Nickel(II), Tau protein, Complex, Potentiometry, UV-Vis and CD spectroscopy

## Abstract

Tau protein plays a crutial role in stablizing the neuronal microtubules in the human brain, under normal physiological environment. This protein contains 12 histidines and 2 cysteines, which are typically the primary metal binding sites. As the metal ions may participate in tau protein aggregation processes and the development of taupathies, we characterized zinc(II) and nickel(II) complexes of tau fragments. The studied peptides were; single-His299 containing tau(292–301) (Ac-GSKDNIKHVP-NH_2_) and its asparagine mutant at position 300, tau(288–293) (Ac-QSKCGS-NH_2_) and tau(289–292) (Ac-SKCG-NH_2_) containing only Cys291, as well as tau(289-V300N) (Ac-SKCGSKDNIKHN-NH_2_) containing both Cys291 and His299. The pH potentiometric, UV-visible and CD spectroscopic measurements allowed us to understand the effect of these side chains on the stability and binding mode of metal ion complexes. For peptides containing a single cysteine and single histidine, nickel(II) complexes with (N^–^,N^–^,N^–^,S^–^) and (N^–^,N^–^,N^–^,N_Im_) coordination modes were main species in alkaline media. UV-visible and CD spectroscopic curves showed that nickel(II) favours the cysteine binding site in the case of the tau(289–300) fragment. Through the bidentate coordination of this tau peptide, the stability of zinc(II) complex is significantly increased compared to fragments containing a single binding site.

**Supplementary Information:**

The online version contains supplementary material available at 10.1007/s12011-026-05162-x.

## Introduction

The total number of people with all-cause dementia worldwide was 50 million in 2010, and this is expected to rise to 113 million by 2050. Over the past 50 years, the incidence of dementia has increased in both high-income and middle/low-income countries due to increased life expectancy [1], while in some high-income countries, the incidence of dementia at a younger age has decreased. This is probably due to changes in education, socio-economic conditions, health care, and lifestyle over the past decades. The incidence of dementia rises sharply after the age of 65 and continues to increase thereafter. The incidence of all-cause dementia is approximately 1 in 100 per year among people aged 65–70, and 1 in 4 per year among people aged 80–90 [2]. 

Approximately 60–80% of all dementia cases are caused by neurodegenerative diseases. Taupathies are a class of neurodegenerative disorders associated with tau protein. The most common and widely studied form of taupathies is Alzheimer’s Disease (AD) [3] which is generally characterized by the loss of cognitive and motor abilities. Its development is concomitant with the accumulation of amyloid-β plaques forming extracellular amyloid plaques, and tau protein aggregating to form intracellular neurofibrillary tangles [4]. The tau protein upon hyperphosphorylation dissociates from the microtubules and oligomerizes to form neurofibrillary tangles in a multistep process [5]. The dyshomeostasis of essential zinc, iron and copper results in increased free metal ions concentration in the neuron triggering the conformational changes in protein and its consequent aggregation majorly by binding to the 2 cysteine or 12 histidine residues present in its sequence, which are the most common primary metal binding sites in tau protein [6, 7]. 

Moreover, several recent studies have focused on how calcium homeostasis may play a role in amyloid beta aggregation and tau hyperphosphorylation processes. It has been shown that disruption of Ca^2+^ homeostasis is an early event in neurodegenerative diseases underlying the cytotoxicity induced by misfolded Aβ aggregates and hyperphosphorylated tau [8]. Calcium concentration is also a very important determinant of the conformational structure of calcium-binding proteins, which, when interacting with other proteins in the brain, can alter their aggregation properties [9, 10]. Thus, further research into how calcium homeostasis and dyshomeostasis play a role in the development of Alzheimer’s disease may shed light on the potential application of these calcium-binding proteins in the field of AD-related biomedicine [11, 12]. 

Although the tau protein itself provides numerous binding sites for metal ions, it is already known that the formation of tau aggregates is also significantly influenced by the hyperphosphorylation of the tau protein. These hyperphosphorylation processes may also be influenced by the interaction between metal ions and tau protein [13]. In order to understand what leads to pathological changes in tau and how this leads to the development of neurodegenerative processes, further information is needed on the interactions between the protein and metal ions and on the processes induced by metal ions.

Tau is an intracellular protein associated with microtubules, and its interaction with tubulin plays an important role in stabilizing microtubules. The aggregation processes of tau protein are characteristic of many neurodegenerative diseases, including AD. It is accepted that disturbances in metal ion homeostasis and balance also play a role in this process [14, 15]. However, the interaction between metal ions and tau protein has only recently become known, further confirming that metal ions have an effect on tau aggregation. Abnormalities in the balance, metabolism, and transport of metal ions lead to an increase in metal ion concentration. This can result in abnormal interactions between metal ions and proteins, which can induce oxidative stress and promote hyperphosphorylation and misfolding of tau protein [16, 17]. 

As more and more biochemical and medical evidence supports the significant role of metal ions in protein conformational changes and subsequent aggregation, research in this area has become widespread. The tau protein contains 12 histidine and 2 cysteine residues, which serve as potential binding sites for metal ions. In addition, these amino acids are located far apart from each other and are present in different amino acid sequences, resulting in different metal ion affinities for different fragments [14]. Previous studies have focused primarily on the complex formation processes of copper(II), iron(II/III), zinc(II), and aluminum(III) ions [14, 18–22], and we have extended our research to include nickel(II) ions [16, 23]. Nickel ions are generally toxic to humans. However, nickel is a trace element present in the human body, primarily derived from the dietary sources, particularly plant-based foods. Nickel has also been detected in the gastrointestinal lining due to the presence of a bacterium, *Helicobacter pylori*, which naturally contains this metal ion and can be present in the human stomach due to infection [24, 25]. However, in the case of certain genetic disorders or excessive environmental stress, the accumulation of nickel(II) metal ions can be assumed, which can lead to an increase in the concentration of nickel(II) metal ions in the human body. In addition, a comprehensive study found high concentrations of nickel(II) metal ions in biological samples taken from patients with neurodegenerative diseases from different geographical locations [26]. 

In our previous studies, we examined in detail the complex formation, redox, and hydrolytic processes of tau fragments containing histidine in different environments in the presence of zinc(II), copper(II)and nickel(II) [16, 21–23, 27]. Our recent studies focused on the 288–300 fragment of tau, which contains both cysteine and histidine. Since both the histidine and cysteine side chains are important binding sites for the Ni(II) and Zn(II) ion, these metal ions can promote or inhibit aggregation processes [28]. 

**Fig. 1:**
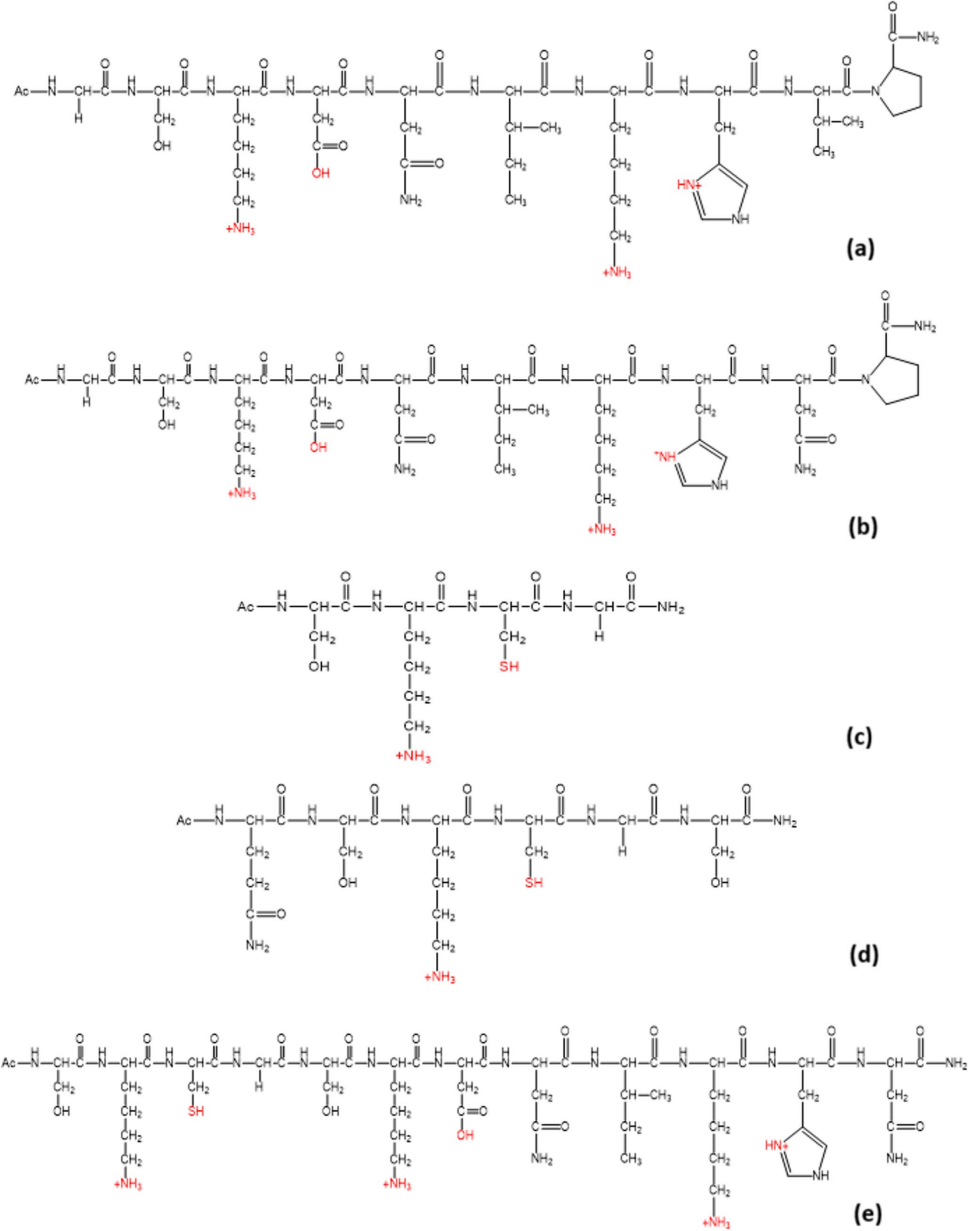
The structural formulae of the studied tau fragments: tau(292-301); Ac-GSKDNIKHVP-NH2 (L1) (**a**), tau(292-V300N-301); Ac-GSKDNIKHNP-NH2 (L2) (**b**), tau(289-292); Ac-SKCG-NH2 (L3) (**c**), tau(288-293); Ac-QSKCGS-NH2, (L4) (**d**), tau(289-300); Ac-SKCGSKDNIKHN-NH2 (L5) (**e**)

We continued our research by studies of Ni(II) and Zn(II) complexes of tau fragments containing the histidine and/or cysteine sites. The studied peptides were tau(289-V300N) (Ac-SKCGSKDNIKHN-NH_2_, L5) containing Cys291 and His299 and tau(288–293) (Ac-QSKCGS-NH_2_, L4) and tau(289–292) (Ac-SKCG-NH_2_, L3) containing only Cys291. The tau(292–301) (Ac-GSKDNIKHVP-NH_2_, L1) and its asparagine mutant at position 300 (L2) were also synthesized and purified for these studies. Our goal was to understand how these side-chain donor groups affect the stability of metal ion complexes and the binding preferences of metal ions. We also analyzed the effect of asparagine, a non-coordinated amino acid similar to valine, in the mutant fragment. The structural formulae of the studied tau ligands are shown in Fig. 1.

## Materials and Methods

### Materials

Chemicals and solvents used for the synthesis of peptides were obtained from the commercial resources in the highest available purity and used without any further purification. The Rink Amide AM resin and all of the N-fluorenylmethoxycarbonyl (Fmoc)-protected amino acids (Fmoc-His(Trt)-OH, Fmoc-Ile-OH, Fmoc-Asn(Trt)-OH, Fmoc-Gly-OH, Fmoc-Lys(Boc)-OH, Fmoc-Pro-OH) were purchased from Novabiochem (Switzerland). The N-methyl-pyrrolidone (NMP), 2-methyl-2-butanol, N-hydroxybenzotriazole hydrate (HOBt∙H_2_O), N,N, N′,N′-Tetra-methyl-o-(1 H-benzotriazol-1-yl)uronium hexafluorophosphate (HBTU), 2,2′-(ethylenedioxy)diethanethiol (DODT) and triisopropyl-silane (TIS) were procured from Merck, while N,N-diisopropylethylamine (DIPEA) and trifluoroacetic acid (TFA) were obtained from Merck Millipore Co. products. Peptide-synthesis grade N,N-dimethyl-formamide (DMF) and acetic anhydride were obtained from VWR International, while dichloromethane, piperidine, acetic acid, diethyl ether and acetonitrile from Molar Chemicals Ltd. All the peptides were in N-acetylated and C-amidated form to remove any end-charge effect.

The cysteine containing ligands tau(288–293) (Ac-QSKCGS-NH_2_) and tau(289–292) (Ac-SKCG-NH_2_) were purchased from Synpeptide Co. Ltd.

The stock solution of NiCl_2_ and ZnCl_2_ were prepared from the analytical grade reagents (Reanal Laborvegyszer Ltd) and their concentrations were determined gravimetrically through the precipitation of oxinate or complexometric titrations.

### MW-Assisted Solid Phase Peptide Synthesis (SPPS) and Purification

The tau(289-V300N) (Ac-SKCGSKDNIKHN-NH_2,_), tau(292–301) (Ac-GSKDNIKHVP-NH_2_) and its asparagine mutant (Ac-GSKDNIKHNP-NH_2_) were synthesized by solid phase peptide synthesis using a microwave-assisted Liberty Peptide Synthesizer (CEM, Matthews, NC). Fmoc/tBut method and HOBt/TBTU/DIPEA activation scheme was used for all these synthesized peptide fragments. Cleaving of α-amino protecting group of the attached amino acids and the amide rink resin was achieved with a 20% V/ V piperidine and 0.1 M HOBt⋅H_2_O in DMF solution at 80 °C with 30 W microwave power for 180 s. The 30 W microwave power for 300 s and the four times excess of amino acids were used for coupling at 80 °C in the presence of a 0.5 M HOBt and 0.5 M TBTU solutions in DMF as an activator and 2 M DIPEA in NMP as an activator base. The free amino terminal was treated manually with DMF followed by 10 ml of acetic anhydride to obtain the acetylated amino group. After the completion of the synthesis the peptides were cleaved from the resin with the simultaneous removal of the side chain protective groups, by treatment with a mixture of TFA/TIS/H_2_O/DODT in 94/2.5/2.5/1% V/V for 2 h. at room temperature. The solution mixture of free peptide fragments was separated from the resin through filtration. The cold diethyl ether precipitated the crude peptide from the solution and washed the contaminants of any reagent during the synthesis or cleaving agents. After performing the filtration, the product was vacuum dried [29–31]. 

The purity of the synthesized peptide fragments was checked by MALDI-TOF MS analysis and furthermore, by analytical RP-HPLC via Jasco instrument, equipped with a Jasco MD-2010 plus multiwavelength detector. We used the following chromatographic settings: Column: Teknokroma Europa Protein C18 (250 × 4.6 mm, 300 Å pore size, 5 μm particle size); elution: gradient elution was performed by solvent A (0.1% TFA in water) and solvent B (0.1% TFA in acetonitrile) at a flow rate of 1 ml/min, monitoring the absorbance at 222 nm [26]. The obtained crude product was further purified using a preparative HPLC to obtain a pure (∼90%) peptide fragment which was finally lyophilized [32–34]. 

### Mass Spectrometry

The purity and identity of the synthesized ligands were verified by MS measurements. For MS measurements, a MicroTOF-Q type Qq-TOF MS instrument (Bruker Daltonik, Bremen, Germany) was used in a positive ion mode. The instrument was equipped with an electrospray ion source with a spray voltage of 4 kV. In the MS/MS experiments, nitrogen was used as the collision gas and the pressure in the collision cell was 1.2 × 10^–2^ mbar. For MS/MS the precursor ions were picked with an isolation width of 5 m/z units. The mass spectra were recorded using a digitizer at a sampling rate of 2 GHz. The mass spectra were externally calibrated using the exact masses of [(NaTFA)_n_ + Na]^+^ clusters produced from the electro-sprayed solution of sodium trifluoroacetate. The sample solutions were introduced directly into the ESI source with a syringe pump (Cole-Parmer Ins. Co., Vernon Hills, IL, USA) at a flow rate of 3 µl.min^–1^. The spectra were evaluated with the Compass Isotope Patern and DataAnalysis 3.4 software from Bruker [35]. The purity of synthesized fragments was confirmed as the MS spectra showed the peaks with molecular mass of the fragment with an accuracy of 4 ppm.

### Potentiometric Measurements

The pH-potentiometric titrations of ligands and their complexes were performed on 3 ml samples at 1 mM and 3 mM total ligand concentration for Ac-GSKDNIKHVP-NH_2_, Ac-GSKDNIKHNP-NH_2_, Ac-SKCG-NH_2_, Ac-QSKCG-NH_2_ and Ac-SKCGSKDNIKHN-NH_2_ respectively, using a carbonate free stock solution of a known concentration of potassium hydroxide (∼0.2 M). The selected metal to ligand concentration ratios were 1:1, 1:2 and 1:3. To ensure the absence of carbon dioxide and oxygen during the titrations, argon gas was continuously bubbled through the sample solution. The samples were being stirred using a VELP scientific magnetic stirrer.

All of these potentiometric titrations were carried out at 298 K and at constant ionic strength of 0.2 M KCl. The pH measurements were registered with a MOLSPIN pH-meter equipped with a 6.0234.100 and 6.0234.110 combination glass electrode (Metrohm) and the titrant gradually added via a computer controlled MOL-ACS burette. While performing the titrations, the equilibrium conditions used were as follows: the system was in equilibrium if the maximum change of pH is 0.001 unit for 30 s. The recorded pH readings were converted into the concentration of hydrogen ion by the method reported by Irving et al. [36] Protonation constants of the ligands and overall stability constants (log*β*_pqr_) of the metal complexes were calculated with the common computational programs (SUPERQUAD [37] and PSEQUAD [38]) based on the Eqs. (1) and (2).

1 $$\mathrm{pM}\;+\;\mathrm{qH}\;+\;\mathrm{rL}\;\rightleftharpoons\;{\mathrm M}_{\mathrm p}{\mathrm H}_{\mathrm q}{\mathrm L}_{\mathrm r}\;\;$$

2 $${\mathrm\beta}_{\mathrm{pqr}}=\:\frac{\left[{\mathrm M}_{\mathrm p\:}{\mathrm H}_{\mathrm q}\:{\mathrm L}_{\mathrm r}\right]}{\left[\mathrm M\right]^{\mathrm p}\:\left[\mathrm H\right]^{\mathrm q}\:\left[\mathrm L\right]^{\mathrm r}}$$

## Spectroscopic Measurements

### UV-Vis Spectroscopy

The similar concentration ratios used for the pH-potentiometry was selected to record the UV-Vis spectra of the nickel(II) complexes. The measurements were performed using a VWR UV1600 PC scanning spectrophotometer at a wavelength range of 200–900 nm.

### Circular Dichroism (CD) Spectroscopy

The circular dichroism (CD) measurements were carried out using a JASCO J-810 spectropolarimeter. The CD spectra of nickel(II) complexes were registered from 220 to 800 nm using 1 cm cell and at the 1:1 concentration ratio used for the pH-potentiometric measurements.

## Results and Discussion

### Acid-Base Properties of Ligands

The protonation constants (log*β*) and deprotonation constants (p*K*) of all the ligands have been determined by SUPERQUAD [37] using the results of potentiometric titrations (Figure S1 in supplementary material) and the values calculated for the tau peptides are reported in Table 1.

**Table 1:** Stability constants (log*β*) and deprotonation constants (p*K*) of fragments from the tau 288–301 region (T = 298 K, I = 0.2 M (KCl))

	L1	L2	L3	L4	L5
log*β* [LH]	10.73 (1)	10.67 (2)	10.33 (4)	10.31 (2)	10.80 (5)
log*β* [LH_2_]	20.74 (1)	20.68 (2)	18.64 (5)	18.49 (3)	21.28 (4)
log*β* [LH_3_]	27.10 (2)	27.04 (2)	ࣧ	ࣧ	31.12 (7)
log*β* [LH_4_]	30.63 (2)	30.54 (3)	ࣧ	ࣧ	39.17 (9)
log*β* [LH_5_]	ࣧ	ࣧ	ࣧ	ࣧ	45.43 (10)
log*β* [LH_6_]	ࣧ	ࣧ	ࣧ	ࣧ	48.95 (10)
p*K* (Asp)	3.53	3.50	ࣧ	ࣧ	3.52
p*K* (His)	6.36	6.36	ࣧ	ࣧ	6.26
p*K* (Cys)	ࣧ	ࣧ	8.31	8.18	8.05
p*K*_1_ (Lys)	10.01	10.01	10.33	10.31	9.84
p*K*_2_ (Lys)	10.73	10.67	ࣧ	ࣧ	10.48
p*K*_3_ (Lys)	ࣧ	ࣧ	ࣧ	ࣧ	10.80

The fully protonated L1 and L2 peptides contain four deprotonation sites: one on the aspartic acid carboxyl group, one on the imidazolium-N of histidine, and two on the two lysine ammonium groups. The L3 and L4 peptides also contain in addition to the thiol group a lysyl side chain. The ammonium group of the lysyl side chain has the highest basicity in all cases, while the deprotonation of the imidazole-N donors occurs at around pH ∼ 6 for both L1 and L2. For the L3 and L4 peptides, the pK value for the thiol group is above 8. In the case of the L5 ligand, there are six protonable donor groups: the COOH group of aspartic acid, the thiol group (-SH) of cysteine, the imidazolium-N of histidine, and the ammonium groups of the three lysine side chains. In this case, the imidazole group of histidine and the thiol group of cysteine are deprotonated in an overlapping step, similar to other peptides containing histidine and cysteine [39, 40]. All these measured values are corresponding with those of other peptides containing aspartic acid, histidine and lysine or cysteine [41–43]. 

## Ni(II) Complexes

### Potentiometry

We performed potentiometric measurements for Ni(II) complexes formed with all the five tau fragments at two M:L concentration ratios. The titration curves of the equimolar solutions are shown in Figure S2 in the supplementary material. All the base volume addition steps in these titrations were fast enough to reach an equilibrium in less than 5 min so no further kinetic studies were planned. As the titration progressed, we observed that the colourless solution turned pale yellow above pH 7 and then became increasingly darker yellow. This is occured because Ni(II) complexes formed in the solution. It can also be seen that the titration curves are shifted to the right as compared to the titration curve of the free ligand. This suggests that this was caused by some kind of extra deprotonation, base-consuming process. It is known from the literature that Ni(II) ions promote the deprotonation and subsequent coordination of amide nitrogen, while a proton is released [44]. This is neutralized by the base. In the case of the Ni:L 1:1 ratio system, 3 extra equivalents of titrant were consumed compared to the p*K* curve (see Figure S1), resulting in the deprotonation of 3 amide nitrogens. Even in the case of an excess of ligand, a proportional shift occurs. At low pH, Ni(II) complexes with an octahedral geometry are present, but as the pH value increases, more and more amide nitrogen is deprotonated, which coordinates to the metal ion and forms Ni(II) complexes with a planar square geometry. This results in a characteristic colour change of solution [45]. Based on our knowledge and the shape of the titration curve, it can be assumed that deprotonation and coordination of the aspartic acid, histidine and/or cysteine side chain takes place in the pH range 3–7, while above pH 7 the amide nitrogens are deprotonated and coordinated.

The stoichiometry and stability constant of Ni(II) complexes were determined from the potentiometric titration data using the PSEQAUD program [38], , which are summarized in Table 2. The data show that complexes with a 1:1 composition and different degrees of protonation are formed.

**Table 2:** Stability constants (logβ) of Ni(II) complexes of fragments from the tau 288–301 region (T = 298 K, I = 0.2 M (KCl))

log*β*	L1	L2	L3	L4	L5
[NiLH_4_]	-	ࣧ	ࣧ	ࣧ	42.22 (10)
[NiLH_3_]	ࣧ	ࣧ	ࣧ	ࣧ	34.93 (8)
[NiLH_2_]	22.85 (43)	23.61 (6)	-	ࣧ	ࣧ
[NiLH]	ࣧ	ࣧ	13.57 (6)	ࣧ	20.58 (3)
[NiL]	ࣧ	ࣧ	ࣧ	ࣧ	12.14 (4)
[NiLH_−1_]	−2.48 (3)	−1.64 (3)	−0.93 (1)	−1.17 (5)	1.92 (4)
[NiLH_−2_]	−13.06 (5)	−11.99 (5)	−8.51 (1)	−8.29 (1)	−9.15 (4)
[NiLH_−3_]	−24.18 (4)	−22.40 (3)	−18.60 (2)	−18.78 (3)	ࣧ
p*K*_12 amide_^a^	ࣧ	ࣧ	7.25	ࣧ	7.18
p*K*_3 amide_^b^	ࣧ	ࣧ	7.58	7.12	8.44
p*K*_13 amide_^c^	8.44	8.42	7.36	ࣧ	7.60
p*K*_1 Lys_^d^	10.58	10.35	10.09	10.49	10.22
p*K*_2 Lys_^e^	11.12	10.41	ࣧ	ࣧ	11.08

^a^ p*K*_12 amide_ = (log *β*[NiLH_*x*_] – log *β*[NiLH_*x*–2_])/2 (*x* = 1 for L3, *x* = 3 for L5)

^b^p*K*_3 amide_ = log *β*[NiLH_*x*_] – log *β*[NiLH_*x*–1_] (*x* = − 1 for L3 and L4, *x* = 1 for L5)

^c^p*K*_13 amide_ = (log *β*[NiLH_*x*_] – log *β*[NiLH_*x*–3_])/3 (*x* = 2 for L1 and L2, *x* = 1 for L3, *x* = 3 for L5)

^d^ p*K*_1_ Lys = log *β*[NiLH_*x*_] – log *β*[NiLH_*x*–1_] (*x* = − 1 for L1 and L2, *x* = − 2 for L3 and L4, *x* = 0 for L5)

^e^ p*K*_2_ Lys = log *β*[NiLH_*x*_] – log *β*[NiLH_*x*–1_] (*x* = − 2 for L1 and L2, *x* = − 1 for L5)

**Fig. 2:**
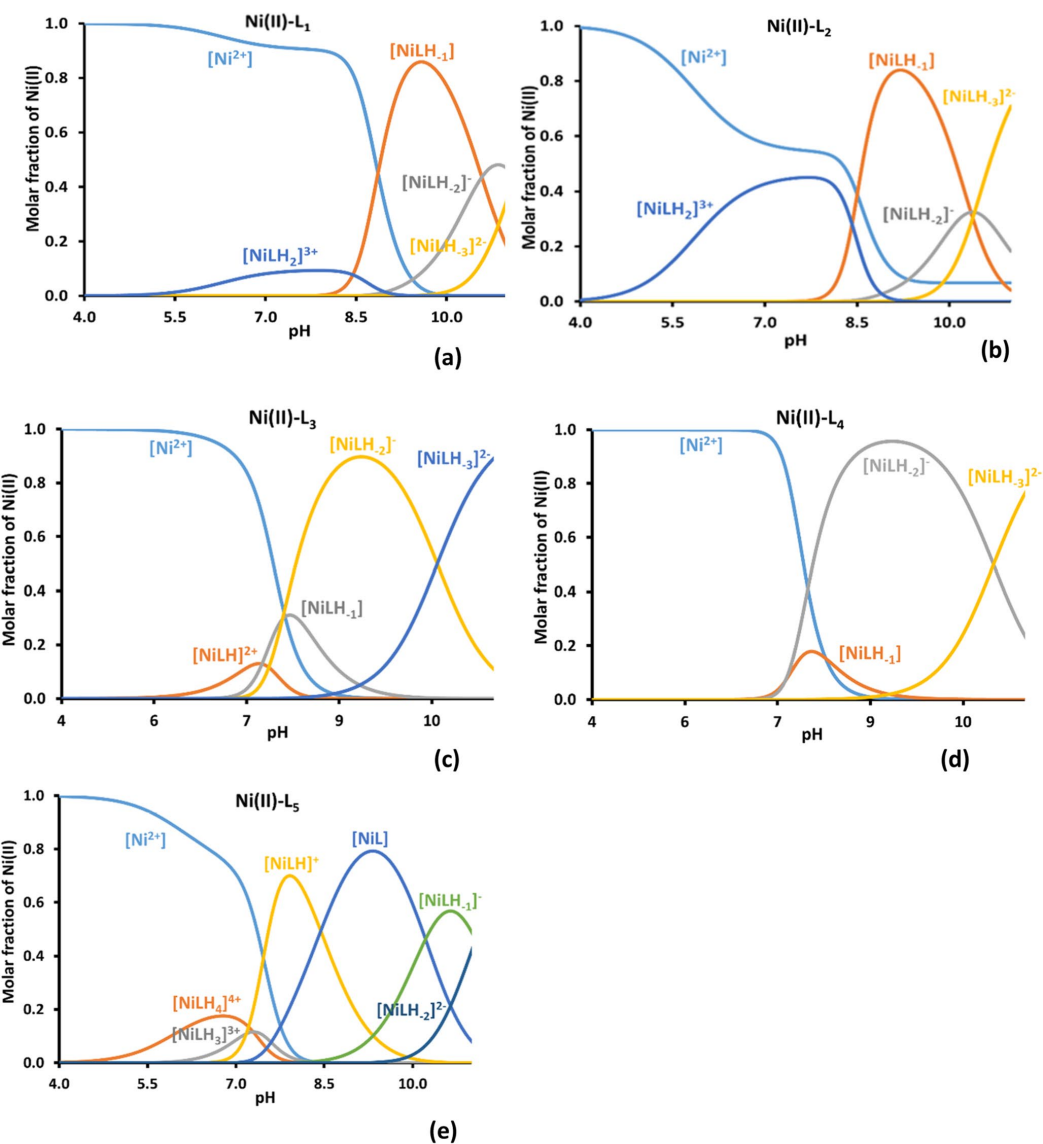
Species distribution diagram of Ni(II) complexes of L1 (**a**), L2 (**b**), L3 (**c**), L4 (**d**) and L5 (**e**) systems in equimolar samples (c(L) = 2 mM)

Based on the stability constants of Ni(II) complexes of ligands L1, L2, L3, L4, and L5, we prepared speciation diagrams using the MEDUSA [46, 47] program, which are shown in Fig. 2. The curves for the Ni(II):L = 1:1 and 1:2 or 1:3 systems were similar to each other, so we present the distribution curves for the equimolar solutions of each of the five tau fragments. The figures clearly show that the complexes form above pH 4 and that different protonated species occur in a slightly acidic medium. In slightly acidic samples, protonated complexes are present, where the L1, L2, L3, and L4 ligands are bound to the metal ion with monodentate coordination (via imidazole or thiolate donor groups), while the lysyl side chain(s) are protonated. ([NiLH_2_] for L1 and L2, [NiLH] for L4 peptide). In the case of the L5 ligand, both mono- and bidentate-coordination are possible via the imidazole and/or thiolate group, while the other groups are protonated. This results in the formation of the [NiLH_4_] and [NiLH_3_] complexes.

By increasing the pH, the coordinated nickel(II) ion induces the deprotonation of the amide nitrogen. In case of the peptides containing only histidyl residues (L1 and L2) the deprotonation of the three amide groups generally occurs in a cooperative manner. As a consequence, the main species where all the four sites of Ni^2+^ are coordinated is [NiLH_− 1_] where metal ion is bonded to one His imidazole N and 3 deprotonated neighbouring amide nitrogen of the chain ([N^–^,N^–^,N^–^,N_Im_] coordination mode). In the case of the studied L3 and L4 ligands containing cysteine, the nickel(II) ion bound to cysteine thiolate group also induces the deprotonation and coordination of the amide nitrogens from the backbone of the peptide chain (and N-terminal acetamide nitrogen for L3). The main complexes are [NiLH_–2_] and [NiLH_–3_] with [N^–^,N^–^,N^–^,S^–^] coordination, the only difference being in the protonation of lysyl side chain.

In the case of the L5 fragment, which contains both histidinyl and cysteinyl residue, deprotonation of the amide nitrogens also occurs parallel with increasing pH. In this case, however, the metal ion can bind to two potential donor sites: [N^–^,N^–^,N^–^,N_Im_] and [N^–^,N^–^,N^–^,S^–^]. To determine which binding site is preferred for the nickel(II) ion, it is worth analyzing the stability constants on the one hand, and spectral studies can provide evidence for the assumption on the other. Since the stoichiometry of complexes with identical coordination modes are not the same, a direct comparison of stability constants is not possible. Therefore, we can compare the derived constants characteristic of amide nitrogen deprotonation (p*K*_12 amide_, p*K*_3 amide_ and p*K*_13 amide_, Table 2, rows 9–12). A comparison of the data for the nickel(II) complexes L1, L2, and L3, L4 clearly shows that the deprotonation of amide nitrogen is characterized by higher p*K*_amide_ values when imidazole is the anchor group and by lower p*K*_amide_ values when the thiolate group is the anchor group. The p*K*_12 amide_, and p*K*_13 amide_ values for the Ni(II)-L5 complexes are closer to those of L3 and L4, which leads us to conclude that nickel(II) binds to the thiolate group to a greater ratio.

In case of the L5 peptide fragment, the third amide nitrogen deprotonation occurs at a relatively higher pH value (p*K*_3 amide_ = 8.44), which may be due to the deprotonation of the terminal acetamide nitrogen in this step, or the higher proportion of the [N^–^,N^–^,N^–^,N_Im_]-coordinated coordination isomer. UV- UV-Visible and CD spectroscopic data can be used to support this conclusion and to determine the ratio of the formed coordination isomers.

Above pH 10, the the deprotonation of lysyl ammonium group takes place in complexes of all ligands. The p*K* value(s) characteristic of these deprotonation steps (Table 2, last two rows) are close to the p*K*_Lys_ values (see Table 1) of the peptides. This suggests that the deprotonation of the ammonium group (-NH_3_^+^) of the lysyl side chains has no effect on the complex formation processes.

### UV-Visible Spectroscopy

**Fig. 3:**
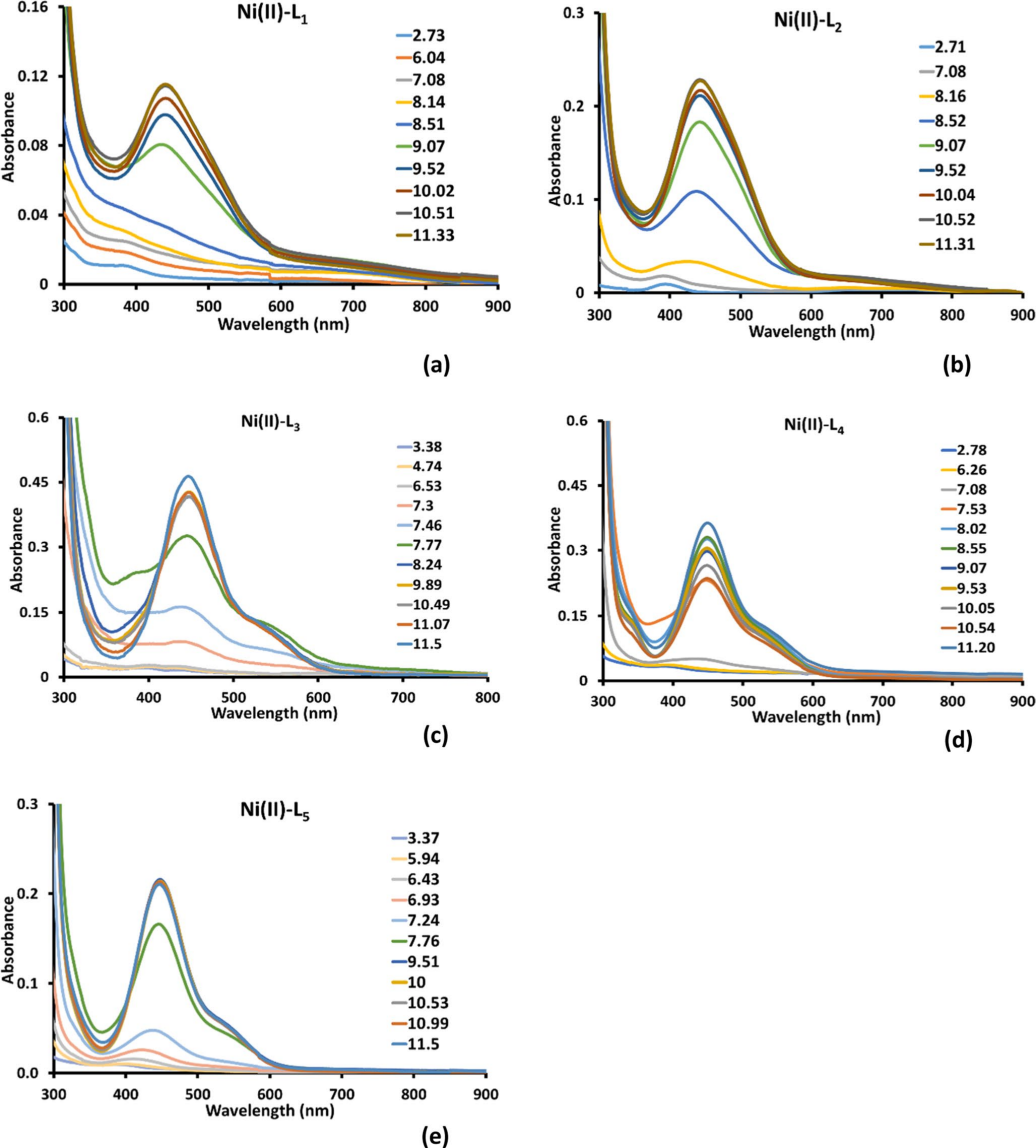
Absorption spectra of Ni(II) complexes of: L1 **(a)**, L2 **(b)**, L3 **(c)**, L4 **(d)** and L5 systems **(e)** at 1:1 ratio (c(L) = 2 mM) in function of pH

The pH-dependent absorption spectra of nickel(II) complexes were recorded between 300 and 900 nm at two metal-ligand ratios (1:3 or 1:2 and 1:1) for all five peptides, and there was virtually no difference between the spectra of the two ratios. Thus, a representative example of these spectra is shown in Fig. 3, which refers to the Ni(II)-L (L = L1-L5) systems obtained at equimolar concentrations. There was practically no change in the nature of the curves or in the intensity when the pH of samples was gradually increased with KOH solution or when the pH of the same sample was restored with hydrochloric acid, indicating that no process other than complex formation was taking place. As can be seen from the figures, the spectra recorded for Ni(II)-ligand systems in the range of pH < 6.5 are of low intensity, which is typical for octahedral complexes of nickel(II) ion [45]. This corresponds to the octahedral geometry of imidazole and/or thiolate coordination complexes appearing above pH 5; however the amide nitrogen and lysines are still protonated and not coordinated.

Above pH ∼7, a new high-intensity band appears around 440 nm for all 5 peptides, supporting the formation of the square-planar Ni(II) complexes with coordination of amide nitrogen [44]. In the case of two cysteine containing L3 and L4 peptides, in addition the high-intensity band characteristic of the square planar complex, a lower-intensity shoulder appears around 540 nm. This is characteristic of thiolate-S-Ni(II) interactions, consistent with the presence of square planar complexes with [N^–^,N^–^,N^–^,S^–^] coordination. Figure S3(a) shows the distribution of species formed in the Ni(II)-L2 system and the absorbance values corresponding to a wavelength of 445 nm as a function of pH, while Figure S3(b) shows the Ni(II)-L3 system in a similar way, but here the absorbance values corresponding to wavelengths of 446 and 540 nm are shown. Both figures confirm that the coordination mode of the Ni(II) complexes produced in the alkaline range is [N^–^,N^–^,N^–^,N_Im_] and [N^–^,N^–^,N^–^,S^–^]., respectively. At very high pH the intensity of the absorption band is constant, so the deprotonation of lysine does not change the coordination sphere of the metal ion.

The difference between the UV-Vis spectra of Ni(II) complexes of L1 and L2 peptides containing histidine and L3 and L4 peptides containing cysteine allows us to conclude the main binding site of Ni(II) in case of the L5 ligand. 

The shoulder appearing around 550 nm in the absorption spectrum (Fig. 3e) and the combined presentation of the speciation curve of nickel(II) complexes and absorption values measured at wavelengths of 448 nm and 543 nm (Fig.S3c) support the conclusion that the N-terminal region containing cysteine is the main binding site for nickel(II). This is consistent with the conclusion drawn from the potentiometric data.

### Circular Dichroism (CD) Spectroscopy

**Fig. 4:**
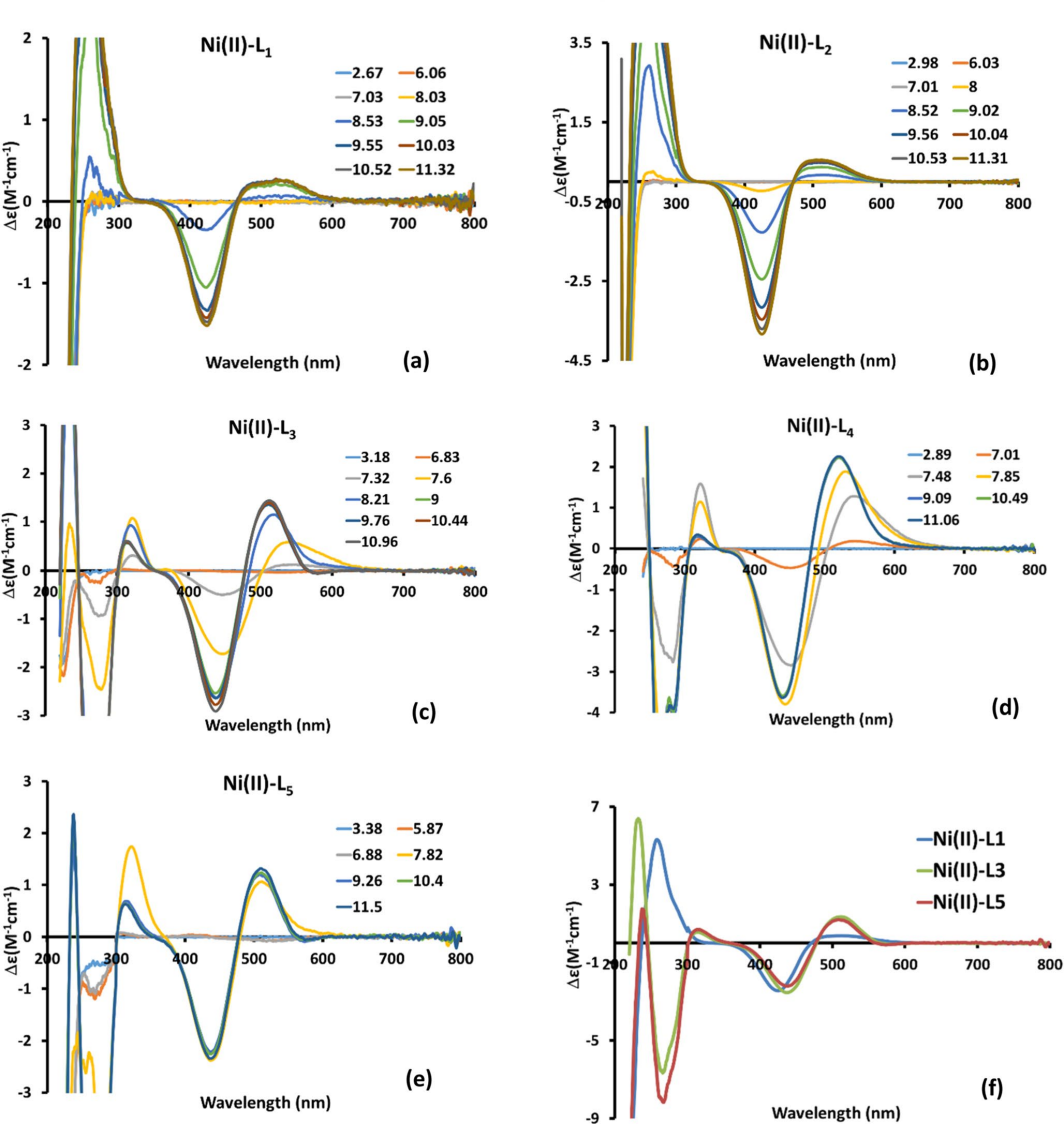
CD spectra of Ni(II) complexes of L1 (**a**), L2 (b), L3 (**c**), L4 (**d**) and L5 (**e**) systems in 1:1 mM solution, at different pH values; comparison of CD spectra recorded at pH 9.0 for Ni(II)-L1, Ni(II)-L3, and Ni(II)-L5 in 1:1 systems (**f**)

CD measurements were performed for all five ligands at a 1:1 Ni:L ratio. The CD spectrum of the complexes was recorded between 800 and 200 nm. The titrant-solution–pH curves obtained in both UV-Vis and CD spectroscopy are consistent with the potentiometric titration curves. This is illustrated in Figure S4 for ligands L2 and L4. No measurable CD activity was observed in slightly acidic samples, which supports the coordination of ligands via the side chain and the presence of octahedral complexes. As the pH value increased above pH 7, the solution became increasingly yellow, and in parallel, both positive and negative peaks appeared in the CD spectrum. The CD-active complexes are due to the formation of planar square complexes through the coordination of the amide nitrogens [45]. This is illustrated in Fig. 4, where the pH-dependent CD spectra of nickel(II) complexes with five ligand systems were recorded at equimolar concentrations.

The positive Cotton effect was detected between 500 and 540 nm, while the negative Cotton effect was detected between 400 and 450 nm. The intensity of the peaks increases with an increase in pH but does not increase further above pH 9. Based on this, we can conclude that the deprotonation and coordination of the three amide nitrogens to the metal ion is not followed by a structural change, and the deprotonation of the lysine residues doesn’t affect the coordination modes of the peptides.

Although the CD spectra of amide nitrogen-coordinated nickel(II) complexes containing histidine and cysteine ligands have similar profiles, differences can be observed in the location and intensity of the Cotton effects. This is further illustrated in Fig. 4f, where the CD spectra of [N^–^,N^–^,N^–^,N_Im_] / [N^–^,N^–^,N^–^,S^–^] coordinated complexes of the histidine-containing L1 and cysteine-containing L4 ligands are compared. The third spectrum in this figure is the CD spectrum recorded in Ni(II)-L5 solution at pH 9. If we compare the latter with the other two CD spectra, it is evident that the CD spectrum of the nickel(II) complex of L5 matches that of L4 (Ac-QSKCGS-NH_2_). This provides further evidence that the N-terminal –SKC– sequence of L5 peptide is the main binding site for Ni(II), and practically all of the metal ions exist in [N^–^,N^–^,N^–^,S^–^]-coordinated complex.

**Fig. 5:**
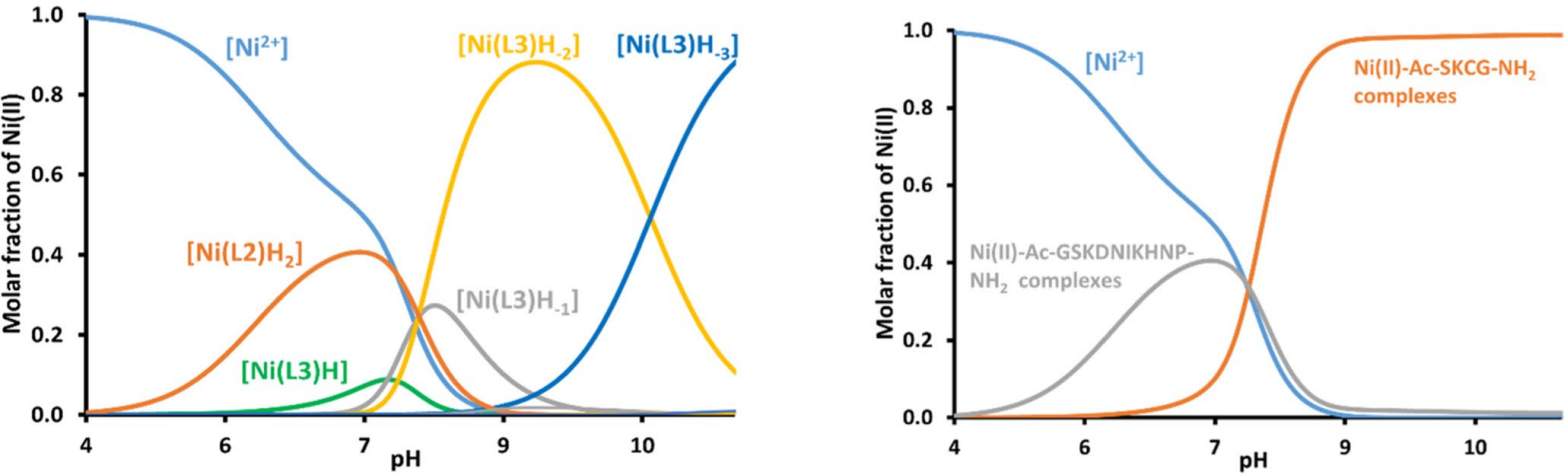
Calculated species distribution diagram of the Ni(II)-L2–L3 system in equimolar solution (c(L) = 2 mM)

**Scheme 1:**
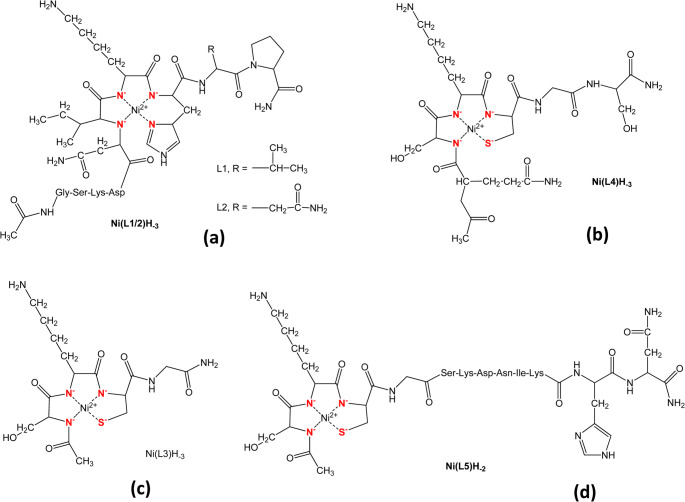
Schematic structural formula of the Ni(Il) complexes of peptides L1 and L2 (**a**), L4 (**b**), L3 (**c**), and L5 (**d**) with [N^–^,N^–^,N^–^,N_Im_] or [N^–^,N^–^,N^–^,S^–^] coordination mode

Our conclusion drawn from potentiometric and spectroscopic measurements is confirmed by the calculated distribution of nickel complexes formed in a Ni(II)-L2-L3 = 1:1:1 system (Fig. 5). In such a solution, the total amount of Ni(II) binds to the L3 peptide, proving that the –SKC– sequence is the binding site for Ni(II) as opposed to the –NIKH– binding site of L2 peptide (Scheme 1).

### Zinc(II) Complexes

We also studied the zinc(II) complexes of the L2 peptide containing one histidine, the L4 peptide containing one cysteine, and the L5 peptide containing both cysteine and histidine. During the titration of samples containing the L2 and L4 ligands with one donor group and zinc(II) ions, precipitation occurred in the alkaline range. During the titration of the solution containing zinc(II) and L5 peptide, no precipitate formed in the pH < 10 range, neither with excess ligand nor at a 1:1 ratio. The stability constants of the Zn(II) complexes are shown in Table 3.

**Table 3:** Stability constants (log*β*) of Zn(II) complexes of fragments from the tau 288–301 region (T = 298 K, I = 0.2 M (KCl))

log*β*	L2	L4	L5
[ZnLH_3_]	-	-	37.24 (2)
[ZnLH_2_]	23.74 (4)	-	28.96 (3)
[ZnLH]	16.47 (6)	14.8 (2)	20.21 (3)
[ZnL]	-	-	10.59 (3)
[ZnL_2_H_2_]	-	30.32 (8)	-
[ZnL_2_H]	-	23.12 (10)	-

In the case of the L2 ligand, only a mono(ligand) complex is formed, in which the metal ion is coordinated by the imidazole nitrogen ([ZnLH_2_]), and carboxyl groups are present in deprotonated forms, while the two lysine groups are protonated. The additional base-consuming process observed during the measurement cannot be attributed to the deprotonation of the lysine groups, as it occurs at a much lower pH (between pH 7 and 7.5, pK for deprotonation step is 7.27) than lysine deprotonation. It means that in the next step, a mixed hydroxide complex forms through the deprotonation of the coordinated water molecule ([ZnLH]), where Zn(II) binds to a hydroxide group in addition to the imidazole nitrogen of the histidyl side chain. The L4 ligand forms mono- and bis-(ligand) complexes of zinc(II) ions, in which the peptide binds via the thiolate group. ([ZnLH], [ZnL_2_H_2_]). Deprotonation of the coordinated water leads to the formation of a mixed hydroxide complex ([ZnL_2_H]). However, in the case of both ligands, only complexes with low stability are formed, which cannot prevent the hydrolysis of the zinc(II) ion and the precipitation of Zn(OH)_2_.

**Fig. 6:**
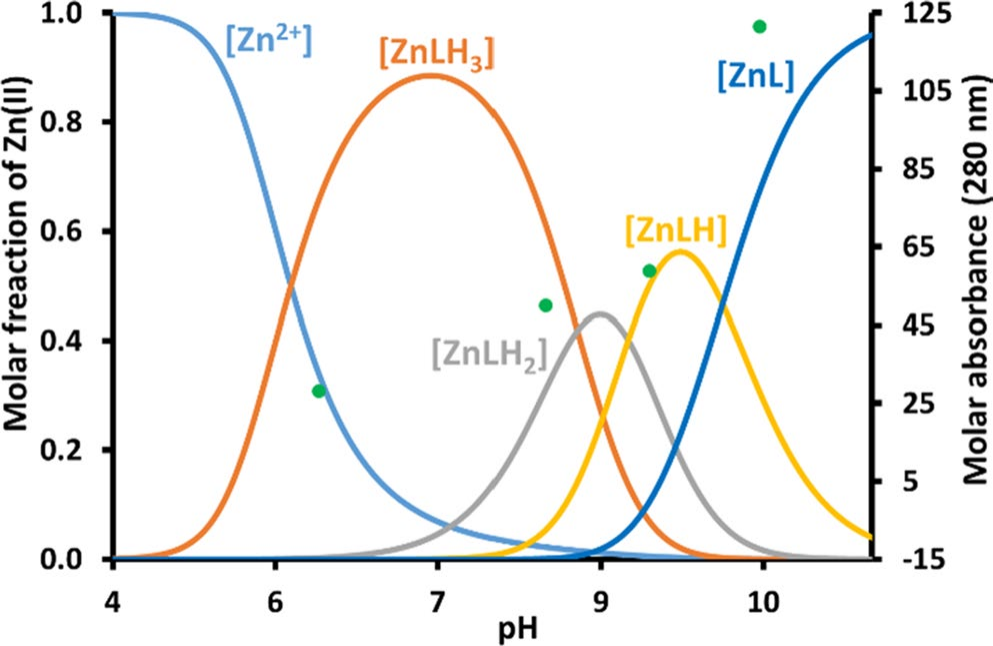
Species distribution diagram of complexes formed in the Zn(II)-L5 system in equimolar solution (c(L) = 2 mM) and the difference between molar absorbance of Zn(II)-L5 and L5 solution (●)

It can be assumed that in the case of Zn(II)-L5, the [ZnLH_3_] ligand is bidentately coordinated, and the three lysyl ammonium groups are protonated. This results in complexes with higher stability than those formed by monodentate coordination of L2 or L4 peptides. The stepwise deprotonation of the [ZnLH_3_] complex results in the formation of [ZnLH_2_] and [ZnLH] complexes, which corresponds to the deprotonation of one or two coordinated water molecules. Presumably, a lysyl ammonium group is deprotonated in the final deprotonation step. Thus, the [ZnLH_2_], [ZnLH] and [ZnL] complexes are mixed hydroxido complexes. The distribution of complexes formed in an equimolar solution of Zn(II)-L5 is shown in Fig. 6. 

The coordination of the zinc ion and the thiolate group was monitored spectrophotometrically. Parallel to the deprotonation of the thiol group, a UV band appears at 280 nm [40]. Thus, spectrophotometric measurements were performed for L5 ligands and Zn-L5 complexes. Figure 6 shows the distribution of zinc(II) complexes and the difference in molar absorbance measured in Zn(II)-L5 and L5 solutions. This shows that, parallel to the formation of zinc complexes, the molar absorbance value is higher than the molar absorbance value of the peptide. This is in agreement with the coordination of Zn(II) ions to the thiolate group.

Figure 6 also clearly shows that complex formation begins in the weakly acidic range, and zinc(II) complexes formed by bidentate coordination of the ligand dominate in the physiological pH range.

**Fig. 7:**
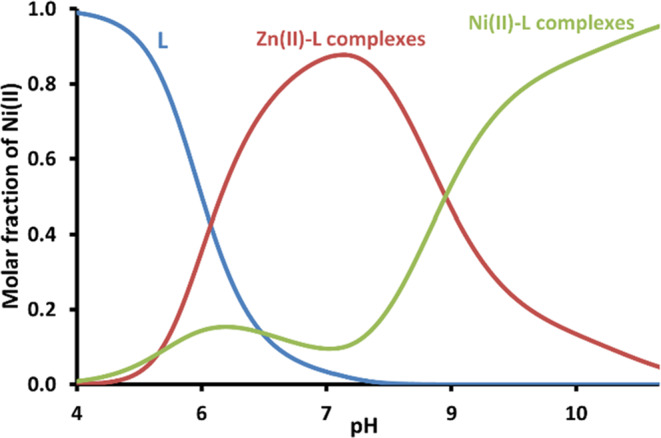
Calculated species distribution diagram of the Ni(II)-Zn(II)–L5 system in equimolar solution (c(L) = 2 mM)

This means that, compared to the complex formation processes of nickel(II), almost all of the zinc(II) ion is present in complex form bound to the tau fragment in the physiological pH range, while in case of nickel(II), this process only becomes significant above pH 7. This difference is even more clearly shown in Fig. 7, which shows the calculated distribution of zinc(II) and nickel(II) parent complexes formed in an equimolar Zn(II)-Ni(II)-L5 solution. At physiological pH, zinc(II) binds to the tau fragment in a much higher proportion than nickel(II) ions. The nickel(II) ion would only be able to displace the zinc(II) ion from the binding sites of the tau fragment at pH values above 8.

## Conclusions

As metal ions may participate in tau protein aggregation processes and the development of taupathies, we investigated the nickel(II) and zinc(II) complexes of tau fragments containing histidine and/or cysteine as potential metal ion binding sites using potentiometric and several spectroscopic (UV-visible, CD) measurements. Tau protein fragments containing the 299 His and 291 Cys environments were synthesized or purchased: these were the following: the histidine-containing tau(292–301) (Ac-GSKDNIKHVP-NH_2_) decapeptide and its Asn mutant, the tau(292-V300N-301) (Ac-GSKDNIKHNP-NH_2_) peptide, the cysteine-containing tau(289–292) (Ac-SKCG-NH_2_) and tau(288–293) (Ac-QSKCGS-NH_2_) fragments, as well as the tau(289-V300N) (Ac-SKCGSKDNIKHN-NH_2_) dodecapeptide containing both binding sites.

The high variability of complex formation processes is due to the presence of histidyl (His299) and/or cysteinyl (Cys291) residues in the sequence, which, in accordance with previous literature results, lead to the formation of mononuclear 1:1 nickel(II) complexes. The complex binding mode is characterized by the coordination of imidazolyl or cysteinyl side chains, as well as the deprotonation and coordination of adjacent peptide nitrogens. The nickel(II) ion is coordinated by [N^–^,N^–^,N^–^,N_Im_] or [N^–^,N^–^,N^–^,S^–^] donors in the main species formed in slightly alkaline solution.

The results showed that replacing valine with asparagine in the tau(292–301) peptide (L1) has a little effect on the stability constant of nickel(II) complexes and does not affect the binding mode of metal ions to the tau fragment. His299 is the main binding site in this tau fragment, and following imidazole coordination, the deprotonation and coordination of the 3 amide nitrogen takes place cooperatively. The deprotonation of the ammonium groups of the two lysyl side chains occurs at higher pH values, but they do not bind to nickel(II) ions.

The studied tau fragments containing cysteine residue form complexes with Ni(II) ions at lower pH values than the histidine-containing fragments. The coordination of nickel(II) ions to thiolate groups also induces cooperative deprotonation of the preceding amide nitrogen, and complexes with [N^–^,N^–^,N^–^,S^–^] coordination dominate in the pH range above 8. This suggests that nickel(II) binds with higher affinity to the thiolate group of cysteine than to the imidazole nitrogen of histidine. Thus, if both binding sites are present in the peptide, the cysteine side chain will be the primary binding site for nickel(II). We have clearly demonstrated this in the case of the tau fragment. Ni(II) binds to the N-terminal –SKC– sequence of the molecule in an equimolar solution of peptide and nickel(II) ion.

Zinc(II) binds to the donor group of the side chains in all five peptides. In the case of ligands L1-L4, the monodentate coordination of the peptides results in complexes with low stability, and their formation doesn’t prevent the precipitation of zinc hydroxide in weakly alkaline pH range. However, the L5 ligand forms zinc(II) complexes with much higher stability through bidentate coordination. These species are present in the form of parent complexes (where only the ligand binds to the zinc(II) ion) and mixed hydroxide complexes (where one or two coordinated water molecules are also deprotonated).

Overall, we can conclude that nickel(II) forms highly stable complexes with fragments of the tau 288–301 region at pH values above 7, and that the preferred binding site for nickel(II) is the sequence containing the amino acid Cys291. However, at physiological pH, zinc(II) binds to the tau fragment in much higher proportions than nickel(II) ions. Thus, zinc(II) ions may be able to prevent the binding of toxic nickel(II) ions to this region of tau protein in physiological pH range. This competition may affect the possible conformational change and aggregation processes of the tau protein. To determine this, we plan to investigate the aggregation of tau fragments under different conditions (in the presence of amyloid-β protein and transition metal ions) in our ongoing research. 

## Supplementary Information

Below is the link to the electronic supplementary material.

Supplementary Material 1

## Data Availability

The original contributions presented in this study are included in the article/supplementary material. The raw data supporting the conclusions of this article will be made available by the authors on request.
