# Supplementary material for: Nickel(II) and Zinc(II) Binding to Tau Protein Fragments Containing Histidine and Cysteine
Source: Biol Trace Elem Res. 2026 Jun 16;204(9):6946–60. doi: 10.1007/s12011-026-05162-x (PMC13437714; doi:10.1007/s12011-026-05162-x)
Supplement: Supplementary file 1 [file 12011_2026_5162_MOESM1_ESM.docx]

**Supplementary Information**

Coordination chemistry of tau fragments containing cysteine and/or histidine

Faareha Mazhar ^1,2^, Noémi Piskolti ^1^, Viktória Kovács ^1^ and Katalin Várnagy ^1,^*

^1^	Department of Inorganic and Analytical Chemistry, University of Debrecen, H-4032 Debrecen, Hungary

^2^	Doctoral School of Chemistry
University of Debrecen
H-4032, Debrecen, Egyetem tér 1, Hungary

*****	Correspondence: varnagy.katalin@science.unideb.hu;

**(a)	(b)**

**Figure S1.** Potentiometric equivalent titration curve of ligands: (cL = 2 mM) (a) L1 and L2, (b) L3, L4, and L5.

**(a)	(b)**

**Figure S2.** Potentiometric titration curves of Ni(II) complexes of ligand systems at equimolar metal to ligand ratios (cL = 2 mM) (a) Titration curve of ligands L1 and L2, (b) Titration curve of L3, L4, and L5.

**(a)	(b)**

**(e)**

**Figure S3.** The percentage distribution curves of the Ni(II) complexes formed at equimolar concentration ratio (c(L) = 2 mM) are plotted together with the change of absorption maxima at: (a)L2 at 445 nm (●), (b) L3 at 446 nm (●) and 540 nm (●) and (c) L5 at 448 nm (●) and 543 nm (●).

**Figure S4.** Potentiometric equivalent curve of Ni(II) complexes of ligands L2 and L4 from potentiometric titrations (solid lines) and titration curve generated from CD spectra (dotted lines)
